# The Relationship Between Pre-pandemic Measures of Religiosity and Psychological Responses to the COVID-19 Pandemic: A Secondary Analysis of Data From a Multi-Country Study

**DOI:** 10.7759/cureus.20013

**Published:** 2021-11-29

**Authors:** Ravi P Rajkumar

**Affiliations:** 1 Psychiatry, Jawaharlal Institute of Postgraduate Medical Education and Research, Puducherry, IND

**Keywords:** urbanization, culture, epidemiology, stress, religiosity, anxiety, depression, covid-19

## Abstract

Background

The uncertainty and socioeconomic disruption caused by the COVID-19 pandemic have been frequently associated with negative affective responses, particularly depression and anxiety. People from countries across the globe have frequently resorted to religious coping to deal with these emotions. However, there are conflicting results in the literature about the impact of prior patterns of religious belief and practice on emotional responses to COVID-19.

Methods

In this cross-sectional, country-level study, the association between pre-pandemic measures of religious affiliation and practice, obtained from prior survey data and self-reported symptoms of depression, anxiety, and stress across 29 countries from a recent multi-country study, were examined while correcting for potential confounders.

Results

There was a trend towards a positive association between pre-pandemic religious belief and practice and anxiety in response to the pandemic (r = .36, p = .057), but this was not significant on multivariate analysis (β = .08, p = .691). Cultural individualism and urbanization were negatively associated with anxiety during the pandemic. There was also preliminary evidence of a non-linear relationship between religiosity and pandemic-related anxiety.

Conclusions

The relationship between religiosity and mental health during the COVID-19 pandemic is unlikely to be a direct one and can be influenced by demographic and cultural factors.

## Introduction

The global coronavirus disease (COVID-19) pandemic, caused by a novel beta-coronavirus (SARS-CoV-2), represents a public health crisis whose scope and impact are without precedent in recent times. Besides the direct effects of the disease in terms of morbidity and mortality, this pandemic has led to widespread social and economic disruptions, as well as abrupt changes in habitual patterns of individual and community behavior. This impact is particularly significant in those who have been required to self-isolate or remain in quarantine for significant periods of time [1,2]. These adverse consequences have been compounded by uncertainty regarding the course of the pandemic, the time frame for economic recovery, and concerns about access to healthcare [3]. During the first wave of the pandemic, experts warned of a potential “tsunami” of global psychological distress and mental disorder, which could include both new-onset symptoms of depression, anxiety, and post-traumatic stress, as well as exacerbations of pre-existing disorders such as obsessive-compulsive disorder and post-traumatic stress disorder [4-6]. Though subsequent research has challenged this generalization, high rates of emergent symptoms of anxiety, depression, and post-traumatic stress have been reported from several nations, particularly in vulnerable groups, such as healthcare or emergency workers [7-9]. There are significant cross-national variations in the prevalence of these symptoms [10].

When interpreting this research, it is important to be aware of certain limitations inherent in the methodologies adopted. Standard screening instruments for depression or anxiety, designed for routine use in clinical or community settings, may not be ideally suited to the unique circumstances of the COVID-19 pandemic. Though pandemic-specific tools have been developed and tested in surveys, their validity and reliability remain debatable [11]. At a higher conceptual level, it is also worth noting that negative affective states, such as depression and anxiety, can be understood as defensive responses to the threat of infection or contagion [12-14]. Seen in this light, simple quantitative measures of depression or anxiety may not adequately distinguish between “adaptive” and “pathological” responses.

The emotional distress triggered by the COVID-19 pandemic has evoked a wide range of coping strategies in affected individuals [15]. One of the most widespread and commonly adopted of these strategies is religious coping [16]. Though beliefs and practices vary significantly according to the specific religion being considered, religious coping itself is almost universal, particularly when a catastrophic or traumatic event affects an entire community [17]. In the context of the COVID-19 pandemic, researchers from several countries have found that religious coping may be associated with lower anxiety, greater optimism, a higher sense of control over one’s circumstances, and increased well-being [18-21]. However, these findings have not been universal. In some cultures, negative religious coping, associated with increased guilt, may predominate [19]. Some particular forms of religion may also be associated with non-adherence to public health measures, such as mask-wearing, social distancing, or immunization [22,23]. Moreover, the widespread restrictions on internal movement adopted by governments in an attempt to curtail COVID-19 transmission have led to restrictions in public religious worship. This may minimize the efficacy of religious coping [24].

As patterns of religious belief and practice are an integral part of a nation or region’s culture and value system, they are deeply entrenched and slow to change. Therefore, it may be possible to assess the impact of religiosity on affective responses to the COVID-19 pandemic by using pre-pandemic data, provided that this data is recent. However, when doing so, it is important to correct for other confounding factors that influence the likelihood of psychological distress during this pandemic. These include existing levels of urbanization and industrialization, regional cultural values such as individualism, economic disruption caused by the pandemic, and the presence of pre-existing psychiatric disorders in individuals [25-29]. If such variables are not taken into account, spurious positive or negative findings may be obtained.

In order to explore the relationship between religious practice and negative affective responses to the COVID-19 pandemic, the following study was designed to examine the relationships between measures of religiosity measured through a global survey from 2018, and estimates of the frequency of depression, anxiety, and stress in response to the pandemic, obtained from a recent multi-national study.

## Materials and methods

This study is an exploratory analysis of the relationship between measures of religious belief and practice at the national level, obtained from the Pew Research Center’s 2018 global survey, and a recent estimate of the rates of depression, anxiety, and stress across 29 countries in response to the COVID-19 pandemic.

Data sources

Depression and Anxiety

Surveys estimating depressive or anxious symptomatology in response to the COVID-19 pandemic have been conducted in over 60 countries [28,29]. Direct comparisons of these estimates are difficult because of differences in the psychometric tools used to measure these symptoms, as well as the cut-off values adopted for “caseness”. In order to overcome this difficulty, the results of a single, recently published, peer-reviewed multi-center study of 35 countries and regions that used a single standard instrument - the 21-item depression, anxiety and stress scale (DASS-21) - to obtain estimates of depression (henceforth abbreviated DEP), anxiety (ANX), and stress (STR) expressed as mean symptom scores, was taken into account. This instrument was selected because of its prior extensive use in patients during the COVID-19 pandemic, its ability to measure three key aspects of psychological distress in this context, and its availability in several languages [30].

Religious Affiliation and Practice

The Pew Research Center’s 2018 publication, “The Age Gap in Religion Around the World”, was used to obtain information on measures of religiosity [31]. This survey, which covered 105 countries, provided percentage estimates of responses to the following questions: religious affiliation (yes/no), attendance at religious services at least weekly (yes/no), daily prayer (yes/no), and whether the respondent considers religion very important in their life (yes/no). The second question was omitted in countries where weekly attendance at religious services is not normative. Responses to these questions were available for 29 of the 35 countries for which DEP and ANX scores were available. These 29 countries were included in the final analysis. The countries included in this study are Argentina, Australia, Brazil, Canada, China, Egypt, Finland, France, Germany, India, Indonesia, Italy, Japan, Malaysia, Mexico, the Netherlands, Nigeria, Pakistan, the Philippines, Portugal, the Republic of Korea (South Korea), the Russian Federation, South Africa, Spain, Sweden, Turkey, the United Kingdom, the United States of America, and Vietnam.

Potential Confounding Factors

Five potential confounding factors were examined in this study. 1) Pre-pandemic prevalence of common mental disorders: Individuals with pre-existing common mental disorders (major depression and anxiety disorders) are more likely to experience an exacerbation of their symptoms during the pandemic, and the prevalence of these disorders has also been observed to vary substantially across countries and cultures [32,33]. Hence, the most recent estimates of the pre-pandemic point prevalence of these disorders (abbreviated GBD (global burden of disease)-DEP and GBD-ANX) for each country were obtained for the year 2019, via a database query from the Global Burden of Disease Collaborative Network [34].

2) COVID-19 indices: Though COVID-19 has affected virtually every nation in the world, there have been marked variations in the prevalence and mortality associated with this illness [35]. It is plausible that countries with a higher caseload or mortality rate may experience higher levels of depression or anxiety. Information on these two parameters expressed as the number of cases (C19 prevalence [C19-PREV]) and the number of deaths per 100,000 population (C19 mortality [C19-MORT]), respectively, was obtained from the Johns Hopkins Medical University’s Coronavirus Resource Center [36].

3) Cultural Individualism/Collectivism: An earlier study of the effect of religious coping on anxiety during the COVID-19 pandemic suggested that this relationship could be moderated by cultural individualism or collectivism [18]. To account for this possibility, each country’s score on the cultural dimension of individualism/collectivism (IC) was obtained from the Hofstede Institute’s database [37]. This score ranges from 0 to 100, with a value of 100 indicating maximal individualism and a score of 0 indicating maximal collectivism.

4) Economic Inequality: While direct measures of the economic disruption caused by the COVID-19 pandemic are difficult to obtain at the international level [38], there is some evidence that this effect is more pronounced in countries or regions with higher pre-pandemic levels of economic inequality [38-40]. Hence, data on the Gini coefficient, a widely used measure of economic inequality that has been studied in the context of COVID-19, was obtained for each country from the World Bank’s database [41].

5) Urban or Rural Residence: A meta-analysis of data on the correlates of anxiety symptoms related to the COVID-19 pandemic found a moderate association between residence in a rural area and higher levels of anxiety. To control for this factor, data on the percentage of the population in each country residing in urban areas, an index of urbanization, was obtained from the World Bank’s database [41].

Data analysis

Data on the mean scores on the DEP, ANX, and STR subscales of the DASS-21 for each country were extracted and tabulated, along with percentages of positive replies to the four questions assessing religious affiliation and practice and values for the confounding variables listed above. All study variables were tested for normality using the Shapiro-Wilk test. Scores for DEP, ANX, and STR all conformed to a roughly normal distribution (Shapiro-Wilk p > .05), as did pre-COVID prevalences of depression and anxiety and measures of cultural individualism. All other variables did not conform to this distribution and underwent natural logarithmic transformation prior to further analysis. Pearson’s bivariate correlation was used to test for linear relationships between measures of religiosity and scores for DEP, ANX, and STR, as well as for interrelationships between these variables and potential confounding factors. These tests were two-tailed, with a significance level of p < .05 considered significant. If significant linear correlations or trends between measures of religiosity and DEP, ANX, or STR were observed, partial correlation analyses were carried out controlling for all confounding variables found significant on bivariate analysis. To examine the effects of these variables on psychological distress as a whole, we also examined the relationships between the total DASS-21 score (the sum of DEP, ANX, and STR, the aggregate of all three abbreviated as DAS) and each of the independent variables.

Variables significantly associated with depression and anxiety in bivariate analyses were entered into a multivariate linear regression analysis, taking depression or anxiety as the dependent variable and the significant correlates as independent variables. A cut-off significance level of p < .1 was used to identify variables included in the multivariate model. No correction was attempted for multiple comparisons in reporting p-values, in view of the small sample size and the exploratory nature of this study.

To minimize the possibility of errors in multivariate analyses, a correlation coefficient of 0.8 or greater on bivariate analysis was taken as indicating multicollinearity between independent variables. As there was a very high degree of correlation between the scores for the items “weekly attendance at religious services”, “praying daily” and “considering religion very important” (r > .9 for all correlations), a composite index of religiosity (REL-COMP) encompassing these three items was computed by taking the arithmetic mean of these three scores for all countries. As the correlation between “religious affiliation” (REL-AFFIL)and the other items was less significant (r = .77, below the threshold for multicollinearity), it was analyzed separately. Similarly, there was a highly significant correlation between C19-PREV and C19-MORT (r = .96); as these two variables could not be meaningfully summed or averaged, the one showing a greater correlation with DEP and/or ANX was included in the multivariate regression where applicable.

In order to explore the possibility of a non-linear relationship between religiosity and mental health outcomes, as has been observed in some pre-pandemic literature on depression [42], the curve estimation function of the Statistical Package for Social Sciences (SPSS) version 20.0 (IBM Corp., Armonk, NY, USA) was used to see if non-linear curve forms could fit the data better than a linear correlation or regression.

## Results

There was significant cross-national variation in mean scores for depression and anxiety across the 29 countries studied. The mean score for DEP was 0.65±0.11, with a maximum of 0.88 in India and a minimum of 0.44 in Nigeria. The mean, score for ANX was 0.48±0.13, with a maximum of 0.84 in India and a minimum of 0.29 in Japan. The mean score for STR was 0.76±0.14, with a range from 1.14 in Egypt to 0.58 in the Netherlands. The mean total score for all forms of psychological distress (DAS) was 1.89±0.34, with a maximum of 2.71 in India and a minimum of 1.38 in the Netherlands.

Bivariate analyses

Table 1 displays the correlation matrix between scores for DEP, ANX, STR, DAS, measures of religiosity (REL-AFFIL and REL-COMP), and other potential confounding factors. Anxiety, depression, and stress were all positively correlated with each other, though the correlation coefficients were below the threshold for suspected multicollinearity, implying that they could be studied individually and not as a single composite factor. No significant correlations were observed between either measure of religiosity and scores for DEP or STR, or for the aggregate score DAS, though there was a trend towards a positive association between REL-COMP and ANX (r = .36, p = .057). Among confounding variables, no significant associations could be found for DEP, while ANX was negatively correlated with urbanization as well as with scores for IC, COVID-19 prevalence, and crude mortality rates. There were trends for a negative association between ANX and pre-pandemic estimated prevalences for depressive disorders (r = -.33, p = .082) and anxiety disorders (r = -.37, p = .052). Stress was negatively correlated with IC but showed no significant associations with other variables. The aggregate score of DAS was negatively correlated with urbanization, but not with any other variable.

**Table 1:** Correlation matrix of associations between pandemic-related levels of depression, anxiety and stress, pre-pandemic measures of religiosity, and other confounding variables. DEP, ANX, STR: Estimated symptom scores for depression, anxiety, and stress that are related to the COVID-19 pandemic; GBD-DEP and GBD-ANX: Estimated prevalence of depressive and anxiety disorders for the year 2019 as per Global Burden of Disease data; REL-AFF and REL-COMP: Measures of religious affiliation and practice for the year 2018 as per Pew Research Survey data; URB: Percentage of population living in urban areas as per the World Bank estimate, 2018; GINI: Gini index of economic inequality as per the World Bank estimate, 2018; C-19-PREV and C-19-MORT: Estimated prevalence and crude mortality rates due to COVID-19 as per the Johns Hopkins data aggregator; LN: Natural logarithmic transformation * significant at p < .05 ** significant at p < .01

Variable	1 DEP	2 ANX	3 STR	4 DAS	5 GBD-DEP	6 GBD-ANX	7 LN REL-AFF	8 LN REL-COMP	9 LN URB	10 LN GINI	11 IC	12 LN C-19-PREV	13 LN C-19- MORT
1	*	.58^**^	.71^**^	.86^**^	.18	.09	.14	.09	-.16	.12	.05	.15	.13
2	-	*	.68^**^	.86^**^	-.33	-.37	.08	.36	-.75^**^	.23	-.45^*^	-.38^*^	-.37^*^
3	-	-	*	.92^**^	-.10	.08	.22	.30	-.35	.21	-.37^*^	-.03	.04
4	-	-	-	*	-.10	-.07	.17	.29	-.48^**^	.21	-.31	-.11	-.08
5	-	-	-		*	.62^**^	.07	-.30	.41^*^	-.10	.56^**^	.53^**^	.54^**^
6	-	-	-		-	*	.15	-.11	.45^*^	-.04	.38^*^	.58^**^	.58^**^
7	-	-	-		-	-	*	.77^**^	-.11	.22	.01	.55^**^	.51^**^
8	-	-	-		-	-	-	*	-.37^*^	.38^*^	-.32	.14	.10
9	-	-	-		-	-	-	-	*	.11	.54^**^	.54^**^	.53^**^
10	-	-	-		-	-	-	-	-	*	-.14	.08	.16
11	-	-	-		-	-	-	-	-	-	*	.51^**^	.50^**^
12	-	-	-		-	-	-	-	-	-	-	*	.96^**^

On examining correlations between measures of religiosity and the other confounding variables included in this study, REL-AFFIL was positively correlated with the estimated prevalence and mortality rates for COVID-19, while REL-COMP was negatively correlated with urbanization and positively correlated with the Gini index of economic inequality.

**Figure 1:**
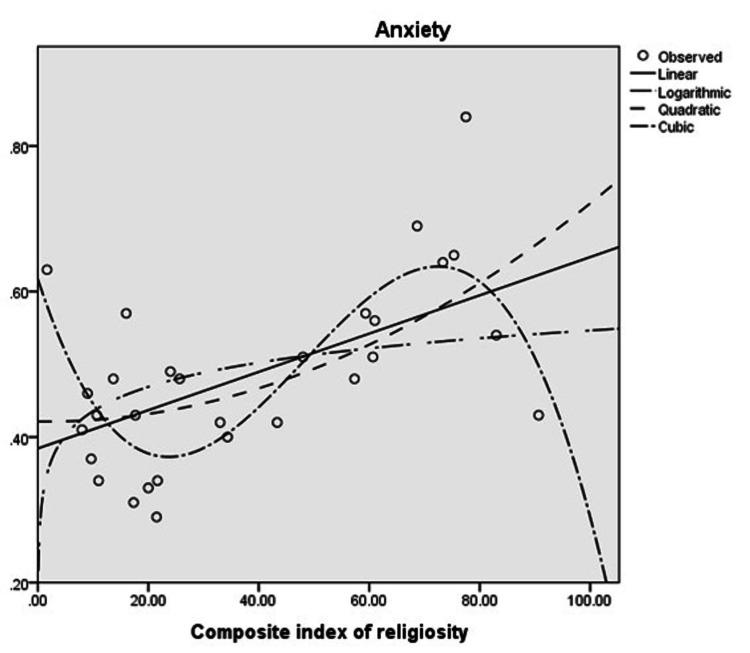
Curve estimation analyses of the relationship between religiosity and pandemic-related anxiety show a possible cubic relationship.

On using the curve estimation function, it was observed that a cubic curve fit the available data on anxiety and religiosity better than linear, logarithmic, or quadratic curves (Figure 1). Using this function explained about 57% of the variance in anxiety (R^2^ for the estimated curve = .565, p = .001). This value was larger than that obtained through linear curve fitting (r = .361, R^2^ = .130, p = .057), but the difference was moderate in size (approximately 43.5%).

Multivariate analyses

In view of the lack of significant bivariate associations for DEP and STR, partial correlation analysis (Pearson’s partial correlation) was carried out only for ANX, examining the link between ANX and REL-COMP while holding the significant variables of urbanization, individualism, and COVID-19 prevalence. In this analysis, the positive trend observed on bivariate analyses is reduced to a non-significant association (partial r = .09, p = .661). Similar results were also obtained when the trend-level variables GBD-ANX and GBD-DEP were also included as covariates (partial r = .09, p = .691)

The results of multivariate linear regression using the enter method, with ANX as the dependent variable, are summarized in Table 2. Independent variables included in the model were GBD-DEP, GBD-ANX, REL-COMP, urbanization, IC, and C19-PREV. The final model attained statistical significance (F = 4.9, p = .003) and accounted for approximately 46% of the variance in ANX (R^2^ = .572; adjusted R^2^ = .455). In this analysis, the only individual variable that was significantly associated with ANX was urbanization, which showed a negative association (β = -.69, p = .003). Variance inflation factors were < 4 for all included variables. When the trend-level variables GBD-DEP and GBD-ANX were removed and the analysis repeated, a similar result was obtained with slightly greater precision (F = 7.98; p < .001; adjusted R^2^ = .50), with urbanization again the only variable remaining significantly associated with ANX (β = -.70, p = .001).

**Table 2:** Multivariate linear regression analyses of pre-pandemic and COVID-19-related variables (independent) associated with anxiety symptom scores (ANX, dependent) in relation to the COVID-19 pandemic ANX: Anxiety symptoms in relation to the COVID-19 pandemic; REL-COMP: Composite measure of religious practice for the year 2018 as per Pew Research Survey data; URB: Percentage of population living in urban areas as per the World Bank estimate, 2018; IC: Hofstede’s index of cultural individualism/collectivism; C-19-PREV: Estimated national prevalence of COVID-19 as per the Johns Hopkins data aggregator; GBD-DEP and GBD-ANX: The estimated prevalence of depressive and anxiety disorders for the year 2019 as per Global Burden of Disease data; LN: Natural logarithmic transformation ** significant at p < .01

Variable	Regression coefficient (β)	Part correlation	Significance level	Variance inflation factor
LN (REL-COMP)	.08	.06	.691	1.85
LN (URB)	-.69^**^	-.47	.003	2.13
IC	-.07	-.05	.741	1.92
LN (C-19-PREV)	.03	.02	.903	2.83
GBD-DEP	.02	.02	.910	2.27
GBD-ANX	-.05	-.04	.797	1.97

## Discussion

Though previous studies have examined the relationship between religiosity and mental health in specific regions during the COVID-19 pandemic [22-26], this study is the first to examine such associations at a cross-national level, while correcting for the effects of confounding variables that could also influence psychological responses to this pandemic.

Among the three outcome variables i.e., depression, anxiety, and stress, only anxiety showed a trend-level positive association with religious belief and practice. This finding suggests that pre-pandemic religiosity may be associated with more anxiety symptoms in response to the pandemic. However, other researchers have obtained different results: some have observed a lack of association between religiosity and anxiety, while others have noted a protective effect against anxiety and some have even identified an increased level of anxiety associated with certain religions in specific contexts [43-45]. The effect of religiosity on psychological responses to the pandemic may depend crucially on factors such as the type of religion being considered, local social and cultural factors, and pandemic-related restrictions on worship [46,47].

A further possibility, as seen above by the results of the curve estimation in Figure 1, is that the relationship between religiosity and pandemic anxiety is non-linear. However, this last finding must be interpreted with caution, as it is based on a small number of data points and could be sensitive to small numbers of outliers at both extremes of the curve. The confirmation of this possibility would require analyses of data from a larger number of countries.

Apart from religiosity, the variables significantly associated with anxiety were COVID-19 prevalence and crude mortality rates, urbanization, and individualism, all of which showed a negative correlation with anxiety during the pandemic. The first of these associations is somewhat counter-intuitive, as during the initial stages of the pandemic, it was expected that countries with a higher load of COVID-19 cases or deaths would experience higher average levels of psychological distress [48]. However, countries with a lower prevalence or fatality rate of COVID-19 may have instituted more stringent control measures, which could lead to higher levels of anxiety [2,49,50]. An association between certain types of anxiety and adherence to protective measures such as hand hygiene and masking has been reported by some authors, suggesting that mild anxiety during a pandemic may be adaptive [27,51]. However, this explanation cannot be confirmed using the current findings.

The association between urbanization and lower levels of anxiety, as well as with a lower total score for depression, anxiety, and stress, is consistent with meta-analyses of community-based research, which have found a significant but modest association between rural residence and the significant symptoms of anxiety [52]. People residing in rural settings, particularly in low- and middle-income countries, may be more likely to experience financial difficulties, shortages of essential supplies during the pandemic, concerns regarding separation from those residing in other regions, and limitations in access to healthcare [53-57]. All of these factors may contribute to the higher levels of psychological distress in countries with a greater percentage of the population residing in rural areas.

A negative correlation between individualism and anxiety was also observed in this analysis. This finding is in contrast to studies of community samples, in which individualistic values predicted poorer psychological adaptation to the pandemic [26,58]. However, they are consistent with an earlier study that suggested that collectivistic cultures were associated with higher levels of alexithymia, which is a predictor of psychological distress [18]. A study of individuals from 26 countries showed that there was no significant association, either positive or negative, between cultural individualism/collectivism and stress related to the COVID-19 pandemic, while another study from a country with a moderate level of individualism showed that individualistic values were associated with active coping [27,59]. The relationship between culture and psychological responses to the pandemic may likely vary depending on the unit of analysis.

Finally, it is worth noting that no association could be found between depression or stress and any of the study variables. While this finding may reflect the methodological limitations discussed below, it may also reflect the fact that a certain degree of depression and stress may be part of the universal behavioral response to a pandemic, and that these forms of distress are only minimally influenced by other factors.

The results of this study are subject to certain limitations, which include the reliance on a single secondary source of data with its inherent methodological limitations such as the underrepresentation of African countries, the use of survey-based measures of religious affiliation and practice which may not capture the symbolic or inner meaning of religious beliefs, the small number of countries included in the analysis relative to the number of variables, the cross-sectional nature of the study, the use of pre-pandemic estimates for the independent variables analyzed in this study, the lack of longitudinal data from each country on the stability of depression, anxiety and stress, and the lack of correction for multiple comparisons on bivariate analyses. In addition, the method adopted in this study cannot distinguish between the effects of different religions, such as Christianity, Hinduism or Islam, each of which have distinct beliefs and practices concerning illness and death. The correction for a large number of potential confounders, in a small sample, may have led to false-negative results in the partial correlation and linear regression analyses. As this study was based on country-level data, its findings cannot be directly extrapolated to individuals. The lack of reliable country-level data on the extent and severity of lockdowns and movement restrictions meant that the association between this variable and mental health could not be examined. Finally, other forms of psychological distress, such as post-traumatic stress symptoms, obsessive-compulsive symptoms, or psychogenic sleep disturbances, were not assessed in this study, though they are also important aspects of the psychological response to COVID-19.

Nevertheless, it is hoped that these findings will be of interest to those examining the links between religiosity, sociocultural factors, and mental health in the context of the COVID-19 pandemic. 

## Conclusions

Despite its limitations, these findings are bound to be of interest to those examining the links between religiosity, sociocultural factors, and mental health in the context of the ongoing COVID-19 pandemic. The possibility of a non-linear relationship between religiosity and anxiety - with low and high levels being a risk factor and intermediate levels being relatively protective - is an intriguing one. But it cannot be taken as definitively established. Studies of individuals from different countries employing more sophisticated statistical methods such as a path analysis examining the links between religious belief and practice, public restrictions of worship, individualism/collectivism, anxiety, and adherence to disease control measures, may confirm or refute the various possibilities considered above, and shed further light on the complex relationship between religious coping and psychological responses to collective trauma.
